# Statistical clumped isotope signatures

**DOI:** 10.1038/srep31947

**Published:** 2016-08-18

**Authors:** T. Röckmann, M. E. Popa, M. C. Krol, M. E. G. Hofmann

**Affiliations:** 1 Institute for Marine and Atmospheric research Utrecht (IMAU), Utrecht University, Utrecht, The Netherlands; 2 Wageningen University, Wageningen, Netherlands; 3 SRON Netherlands Institute for Space Research, Utrecht, Netherlands

## Abstract

High precision measurements of molecules containing more than one heavy isotope may provide novel constraints on element cycles in nature. These so-called clumped isotope signatures are reported relative to the random (stochastic) distribution of heavy isotopes over all available isotopocules of a molecule, which is the conventional reference. When multiple indistinguishable atoms of the same element are present in a molecule, this reference is calculated from the bulk (≈average) isotopic composition of the involved atoms. We show here that this referencing convention leads to apparent negative clumped isotope anomalies (anti-clumping) when the indistinguishable atoms originate from isotopically different populations. Such statistical clumped isotope anomalies must occur in any system where two or more indistinguishable atoms of the same element, but with different isotopic composition, combine in a molecule. The size of the anti-clumping signal is closely related to the difference of the initial isotope ratios of the indistinguishable atoms that have combined. Therefore, a measured statistical clumped isotope anomaly, relative to an expected (e.g. thermodynamical) clumped isotope composition, may allow assessment of the heterogeneity of the isotopic pools of atoms that are the substrate for formation of molecules.

Analysis of the isotopic composition of molecules is one of the key tools for studying element cycles on earth. For the light elements H, C, N and O with relatively small heavy-to-light isotope ratios at natural abundance, the standard analytical instruments have largely limited isotope analysis to single-substituted isotopocules (isotopically substituted molecules). Studies of multiply substituted isotopocules, referred to as clumped isotopes, were only occasionally carried out, often using isotopically enriched substrates or labeling experiments^1^,^2^,^3^,^4^,^5^. Recent analytical advancements using isotope ratio mass spectrometry^6^,^7^,^8^ or laser spectroscopy^9^ have enabled high precision measurements of clumped isotopes in several molecules such as CO_2_, CH_4_, O_2_ and N_2_O^8^,^10^,^11^,^12^,^13^,^14^,^15^ and the field is rapidly expanding.

Since multiply substituted isotopocules are thermodynamically more stable than single substituted ones, classical isotope theory predicts small but measurable positive clumped isotope anomalies for most molecules under natural conditions^16^,^17^,^18^,^19^. These clumped isotope signatures depend on temperature, which is the basis of the new field of clumped isotope thermometry^20^.

Yeung *et al*.^14^ and Wang *et al*.^13^ reported negative heavy isotope clumping in photosynthetic O_2_ formation and in biogenic CH_4_, respectively. Yeung *et al*.^14^ attributed the negative Δ values (see equation 3 for definition) in photosynthetic O_2_ to different isotopic composition of the two O atoms originating from different sites in the oxygen evolving complex of photosystem II. Triggered by this observation we investigated this further and show here that negative clumping anomalies are necessarily expected whenever two or more indistinguishable atoms of the same element but with different isotopic composition combine in a molecule. The atoms do not need to share a common bond but can be at distant places in a molecule.

Yeung^21^ recently presented an analysis of such apparent statistical clumped isotope effects in combination with other isotope effects. In our paper, we restrict the analysis to statistical clumped isotope effects and phrase the calculations exclusively in terms of isotope ratios in order to elucidate the underlying general nature of these apparent isotope signatures. The fundamental origin of the apparent statistical clumped isotope effect is thoroughly presented and visualized geometrically. We then provide a general mathematical formalism for apparent statistical clumped isotope signatures in any multiple isotope system. Finally we demonstrate quantitatively how a certain measured statistical anti-clumping signal can be used to determine the isotopic heterogeneity of indistinguishable atoms in a molecule.

## Origin of the statistical negative clumped isotope signatures

We describe and calculate the statistical clumped isotope effects in terms of heavy-to-light isotope ratios ^i^*R* of individual atoms and molecules, where the index *i* indicates the mass of the atom or molecule. The same letter *R* is used for both atomic isotope ratios and molecular isotopocule ratios. In particular, for molecules with multiple heavy isotopes (clumped isotopes), the heavy-to light isotopocule ratio is defined as


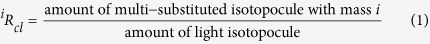


When two atoms with atomic heavy-to-light isotope ratios *R*_1_ and *R*_2_ (where *R*_*i*_ can be, e.g., ^2^H/^1^H, ^13^C/^12^C, ^18^O/^16^O, etc.) combine in a molecule in a purely random manner, i.e., without any isotope effect, the ratio of molecules that include the heavy isotopes of both of these atoms relative to molecules including only light isotopes is simply the product of the atomic isotope ratios of the two atoms


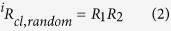


Clumped isotope signatures Δ_i_ are then by convention calculated as the relative difference between a certain (measured) clumped isotopocule ratio and the random clumped isotope ratio, usually reported in per mill (‰).


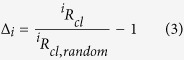


**Figure 1:**
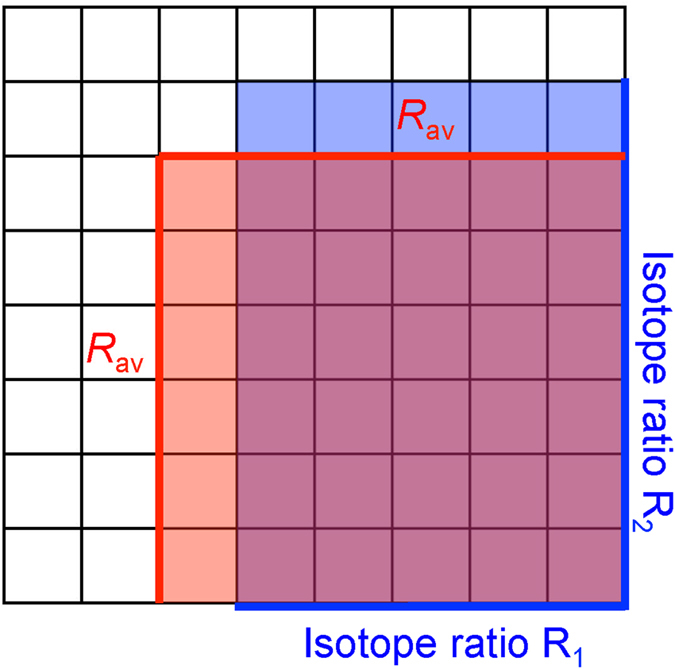
Geometric interpretation of the apparent statistical clumped isotope signatures. The quantity ^*i*^*R*_*cl*,*random*_ that is used as a reference to calculate Δ_i_ (Equation 3) is the product of the individual isotope ratios *R*_1_ and *R*_2_, indicated by the blue lines and blue area. However, for indistinguishable atoms in a molecule the individual ratios *R*_1_ and *R*_2_ cannot be determined independently, and they are both assigned the average ratio *R*_av_ (red lines). This leads to the red area for ^*i*^*R*_*cl*,*random*_. The systematic error associated with using the red area instead of the blue area to calculate Δ_i_ causes the apparent negative clumped isotope signatures described in this paper.

The apparent negative statistical clumped isotope signatures that we describe in this paper are fundamentally related to this referencing convention, in particular the choice of the reference ratio ^*i*^*R*_*cl*,*random*_ that is required to calculate Δ_i_ in Eq. 3. When the isotope ratios *R*_1_ and *R*_2_ of the involved atoms are individually known, ^*i*^*R*_*cl*,*random*_ = *R*_1_ · *R*_2_ can be precisely calculated. This is always the case when heavy isotopes of different elements clump together (e.g. ^13^C and ^18^O in CO or in CO_2_). It also holds when molecules of the same element, for which the individual isotope ratios are known, clump together (e.g. ^15^N^α^ and ^15^N^β^ in N_2_O, where α and β indicate the central and terminal position of the N atom in the linear NNO molecule, which can be determined independently^22^,^23^). This is graphically illustrated in Fig. 1, where *R*_1_ and *R*_2_ are plotted on the x- and y-axis and their product, ^*i*^*R*_*cl*,*random*_, is shown as the blue area.

When a molecule contains indistinguishable atoms of the same element, it is impossible to determine the individual atomic isotope ratios of these atoms. Nevertheless, the atoms may originate from isotopically distinct populations with isotope ratios *R*_1_ and *R*_2_. To calculate the correct value of ^*i*^*R*_*cl*,*random*_ we would therefore need to know the individual isotope ratios *R*_1_ and *R*_2_. However, since these isotope ratios cannot be retrieved for indistinguishable atoms, it is common (and reasonable) to assign the bulk (≈average, see below) isotopic composition of the atoms. Through this choice, the real stochastic clumped isotope ratio *R*_1_ ⋅ *R*_2_ (blue area in Fig. 1) is substituted by the approximated value *R*_*av*_ ⋅ *R*_*av*_ (red area in Fig. 1). Both areas have the same perimeter 2 ⋅ (*R*_1_ + *R*_2_) =2 ⋅ (*R*_*av*_ + *R*_*av*_), but the red square has a larger area than the blue rectangle, i.e., *R*_*av*_ ⋅ *R*_*av*_ > *R*_1_ ⋅ *R*_2_. Replacing the area of the blue rectangle by the one of the red square in the denominator of Eq. 3 causes a systematic negative artifact. This produces the apparent negative statistical clumped isotope effect. The anti-clumping signal is the larger the more different the individual isotope ratios of the indistinguishable atoms are. In the following chapters we derive the general mathematical formalism to calculate these apparent statistical clumped isotope effects in any multi-isotope system. We also show that the measurement of statistical anti-clumping in principle allows quantifying the heterogeneity of the isotopic composition of indistinguishable atoms in a molecule, i.e. to reconstructing the blue area from the red area in Fig. 1.

We emphasize that the apparent anti-clumping signature is not related to a physical isotope effect, but is a mathematical artifact that originates from the referencing convention. It will never occur when the contributing atoms are distinguishable (thus never for atoms from different elements, e.g. for ^13^C-^18^O clumping in CO_2_), but it will always occur when two indistinguishable atoms of the same element combine in a molecule (e.g. for ^18^O-^18^O clumping in CO_2_). Table 1 shows a selection of common atmospheric molecules and specific clumping signatures for which statistical anti-clumping will occur or not occur, respectively.

### Clumping of indistinguishable atoms in one molecule

#### Molecules with two atoms of the same element (e.g. N_2_, O_2_, H_2_)

As mentioned above, two indistinguishable atoms of the same element in one molecule may generally originate from different reservoirs or involve different fractionation effects such that their isotope ratios *R*_*1*_ and *R*_*2*_ represented two distinct pools when the molecule formed. Since the two atoms are now indistinguishable, we cannot independently measure the isotope ratios *R*_*1*_ and *R*_*2*_. In fact, in conventional isotope ratio measurements of single substituted isotopocules the arithmetic average ratio of the two ratios (e.g. ^29^*R* = 2 ^15^*R*_av_ for ^15^N measurements in N_2_) is determined. For rare heavy isotopes (*R*_1_, *R*_2_ ≪1), this average ratio ^15^*R*_*av*_ is generally similar to the bulk isotope ratio *R*_*bulk*_ of the sample (see Supplementary Information). In this case, it is common and reasonable to assign *R*_*bulk*_ ≈ *R*_*av*_ to each of the indistinguishable atoms for further calculations. For the remainder of this paper, we use *R*_*bulk*_ = *R*_*av*_, which considerably simplifies the formulas and removes the dependency of the apparent clumped isotope signal on the isotope ratio. The differences between using *R*_*bulk*_ and *R*_*av*_ are discussed in detail in the Supplementary Information.

The stochastically expected (random) ratio of isotopocules with two heavy atoms relative to the light isotopocules, from a population of atoms with average heavy isotope ratio of 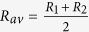, is


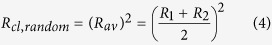


However, when the atoms represent different isotopic pools with possibly different isotope ratios, the real clumped isotope ratio, *R*_*cl*_, of doubly substituted isotopocules relative to the light isotopocules is


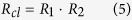


The apparent statistical clumped isotope Δ is the relative difference between the real and the stochastically expected clumped isotope ratio


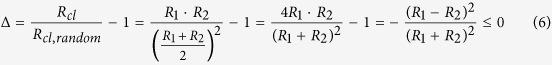


**Figure 2:**
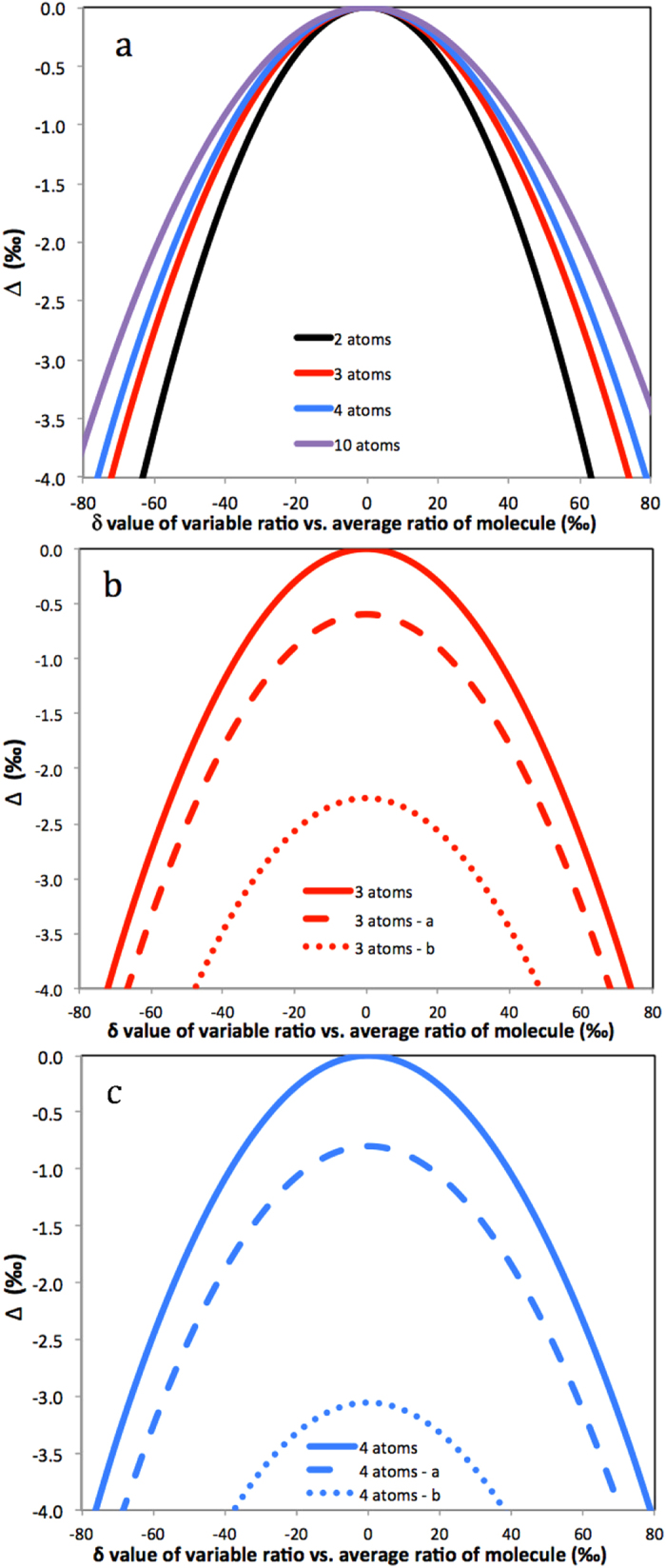
Dependence of the apparent statistical clumped isotope signal for combining two (black), three (red), four (blue) or 10 (purple) indistinguishable atoms in a molecule. In all cases one isotope ratio is varied to produce the relative difference of this ratio from the mean of all isotope ratios (x axis scale). In (**a**) and the solid lines in (**b,c**) the other isotope ratios are fixed and identical. This case includes the situation where all isotope ratios are equal and Δ = 0‰. In (**b**) (for 3 indistinguishable atoms) and (**c**) (for 4 indistinguishable atoms) the solid lines are the same curves as in (**a**). The dashed lines show the case where one of the fixed isotope ratios is increased by 5% relative to the others, and the dotted line shows the case where one isotope ratio is increased by 10%.

The clumped isotope composition is always negative, except for the case *R*_*1*_* *=* R*_*2*_, for which Δ = 0. Thus, when two atoms of the same element with different isotopic composition combine in a molecule, the resulting molecule will always have an apparent negative clumping signature. The black curve in Fig. 2a shows the size of this quadratic statistical negative isotope clumping according to Eq. (6). The negative clumping signal Δ does not depend on the absolute value of the underlying isotope ratios, but only on the relative difference of the isotope ratios. In the following we refer to this effect as “statistical clumped isotope signature”. Eq. 6 was first derived for the case of formation of molecular O_2_ in photosynthesis by Yeung *et al*.^14^, who indeed observed negative clumped isotope signals relative to the thermodynamically expected values for photosynthetic O_2_.

### Generalization: Molecules with three or more atoms of the same element – complete substitution

When a molecule contains three or more atoms of the same element, these atoms can generally represent populations with different isotope ratios *R*_*1*_, *R*_*2*_, … *R*_*n*_. We first consider the case of full heavy-isotope substitution, which is the generalization of the two-atom case presented above. As it is not possible to independently measure the individual isotope ratios *R*_*i*_ of the indistinguishable atoms, the arithmetic average isotope ratio ^18^*R*_*av*_(≈^18^*R*_*bulk*_, see Supplementary Information) is assigned to each of the indistinguishable atoms for further calculations


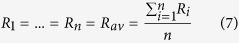


As the atoms are all assigned the same atomic isotope ratio *R*_*av*_, the stochastically expected ratio of fully substituted isotopocules relative to non-substituted isotopocules from this population of atoms is the *n*-th power of *R*_*av*_.


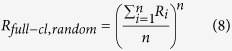


This is the *n*-dimensional equivalent of replacing blue rectangle in Fig. 1 by the red square. The real (=observed) ratio of fully-substituted isotopocules is the product of all isotope ratios involved, which is identical to the *n*-th power of the geometric mean of the isotope ratios


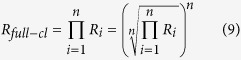


Thus, the statistical clumped isotope signature for fully substituted isotopocules is


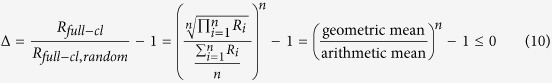


This equation applies to any set of indistinguishable atoms in a molecule. Since the arithmetic mean is always larger or equal than the geometric mean, the statistical clumped isotope signal is always negative, except for the case where all ratios *R*_i_ are identical, in which case the arithmetic and geometric means are equal and thus Δ = 0. Figure 2 shows the variation of Δ with the relative difference of the isotope ratios in the 2-, 3-, 4- and 10-atom systems. For the cases illustrated in Fig. 2a, only one isotope ratio is varied and the isotope ratios of all other atoms are kept constant and identical. For the same relative difference in isotope ratio of a single atom, the clumping signal decreases with increasing number of atoms. Although the 10-atom case may not be of much practical use, it is included to emphasize the point that the statistical negative heavy isotope clumping does not require the heavy isotopes to be linked directly by a common chemical bond. For example, statistical negative D-D clumping in ethane (C_2_H_6_, Table 1) may involve pairs of hydrogen atoms at any of the 6 positions.

The isotope combinations presented in Fig. 2a all include the point where all isotope ratios are equal, which corresponds to Δ = 0‰. However, in general, the isotope ratios of the atoms at different positions are not equal. Figures 2b,c show the clumping signal for the 3- and 4-atom cases when one ratio is varied again, and the other ratios are held constant, but at different values for the individual atoms. Now the situation that all isotope ratios are equal cannot occur and the parabola-shaped curves are shifted towards negative Δ values. The y-axis offset increases with increasing difference of the individual isotope ratios, thus with the heterogeneity of the isotopic composition of the individual indistinguishable atoms. The curves in Fig. 2b,c are selected 2-dimensional cross-sections of a multi-dimensional space, which illustrate the effect of varying one of the multiple isotope ratios relative to a fixed set of other ratios. In practice, a certain combination of isotope ratios among indistinguishable atoms will correspond to one single value of Δ and we will show below that the statistical clumped isotope signal Δ is a measure for the heterogeneity of the isotopic composition of indistinguishable atoms in a molecule.

### Molecules with three or more atoms of the same element – incomplete substitution

#### Clumping of two heavy isotopes in molecules with three indistinguishable atoms (e.g. ^18^O-^18^O or ^17^O-^17^O clumping in O_3_ or NO_3_)

As a first example we consider molecules with three indistinguishable atoms and calculate the clumping signature of double substituted isotopocules. The three atoms generally represent three isotopically different populations with isotope ratios *R*_*1*_, *R*_*2*_ and *R*_*3*_. However, as the atoms are indistinguishable, the individual ratios cannot be determined and for further calculations they are assigned the average isotope ratio, which can be determined from measurement of the single substituted isotopocules


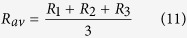


The stochastically expected isotope ratio of isotopocules with exactly two out of three possible heavy isotopes relative to the light isotopocules from a population of indistinguishable atoms with assigned isotope ratio *R*_*av*_ is


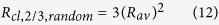


The factor 3 gives the number of possible permutations of two heavy isotopes over three atom positions. The real probability for finding a molecule with exactly two heavy atoms from the three atoms with isotope ratios *R*_*1*_, *R*_*2*_ and *R*_*3*_ is


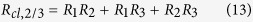


Thus, the statistical clumped isotope signal for clumping of two out of 3 possible heavy isotopes, Δ_2/3_, is


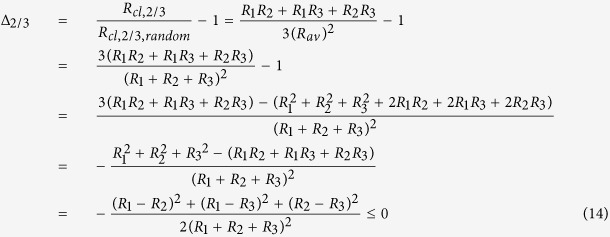


**Figure 3:**
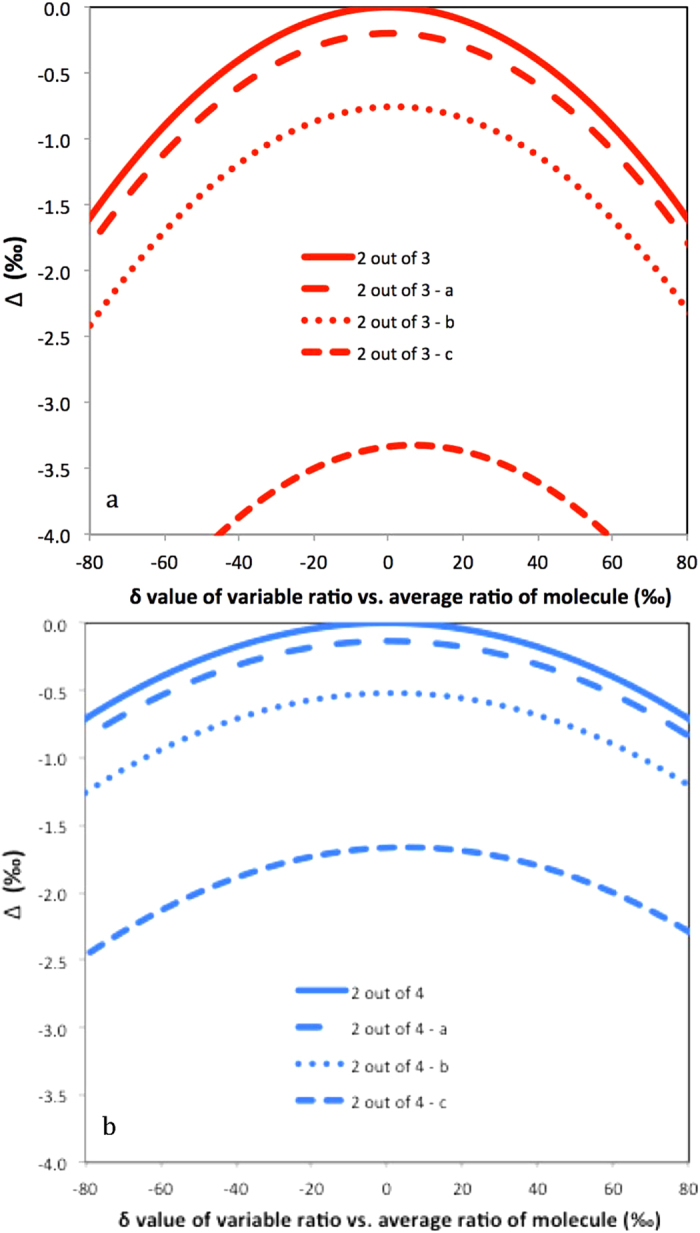
Statistical heavy isotope clumping of two out of three (**a**) and two out of four (**b**) heavy isotopes. In all cases shown, the isotope ratio of one atom is varied relative to the mean of the other ratios as indicated on the x axis and the other ratios are fixed. In the base case (solid line), the isotope ratios of the other atoms are identical. This includes the situation for which all isotope ratios are identical (Δ = 0‰), in case a (dashed line) one of the fixed isotope ratios is increased by 5%, in case b (dotted line) one of the fixed isotope ratios is increased by 10% and in case c (long-dashed line) one of the fixed isotope ratio is increased by 10% and another ratio is decreased by 10%.

Since all terms in the numerator and denominator are squares and thus positive, Δ is always negative except for the case *R*_1_ = *R*_2_ = *R*_3_ when Δ_2/3_ = 0. Some examples for the clumping of two out of three heavy isotopes in a molecule are shown in Fig. 3a. The solid line again includes the case where all ratios are identical and Δ = 0. The dashed and dotted lines show examples where one isotope ratio varies and the other ones are constant but different, so that the case that all are equal does not occur. Again, the dashed and dotted curves are shifted to more negative Δ values.

### Clumping of two heavy isotopes in molecules with four indistinguishable atoms (e.g. D-D clumping in CH_4_)

We now consider molecules with four indistinguishable atoms and calculate the clumping signature of doubly-substituted isotopocules. The four atoms generally represent isotopically distinct pools with isotope ratios *R*_*1*_, *R*_*2*_, *R*_*3*_ and *R*_*4*_. As the ratios cannot be determined individually, they are assigned the average atomic isotope ratio


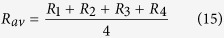


The stochastically expected probability for forming a molecule with exactly two out of four possible heavy isotopes from a population of indistinguishable atoms with assigned isotope ratio *R*_*av*_ is


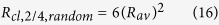


The factor 6 again gives the number of possible permutations of 2 heavy isotopes over 4 atom positions 
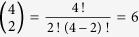
. However, the real probability to form an isotopocule with exactly two heavy atoms from the four atoms with different isotope ratios *R*_*1*_, *R*_*2*_, *R*_*3*_ and *R*_*4*_ is


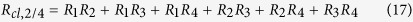


Thus, the clumped isotope signal Δ is


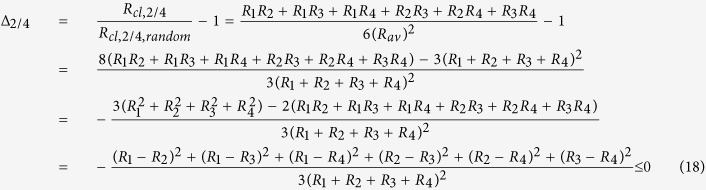


Since Δ can again be expressed as a negative sum of squares, it is always negative, except for *R*_1_ = *R*_2_ = *R*_3_ = *R*_4_ where Δ_2/4_ = 0. Some examples for the statistical clumped isotope effect of two out of four heavy isotopes in a molecule are shown in Fig. 3b. An important example for this case is the D-D clumping in methane. The reservoirs that supply the different hydrogen atoms in the formation of methane can vary considerably and significant apparent statistical anti-clumping is expected.

### Generalization: Clumping of *m* heavy isotopes in molecules with *n* indistinguishable atoms

For the general case of *n* indistinguishable atoms that represent isotopic pools with isotope ratios *R*_*1*_, *R*_*2*_, … *R*_*n*_, we assign again the arithmetic mean isotope ratio *R*_*av*_ to each of the atoms.


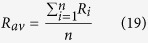


The stochastically expected ratio of isotopocules with exactly *m* out of a possible *n* heavy atoms relative to the light isotopocules from a population of indistinguishable atoms with this average heavy isotope ratio *R*_*av*_ is


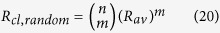


The real ratio of isotopocules with *m* heavy isotopes relative to non-substituted isotopocules (*R*_*cl*_) from *n* atoms with isotope ratios *R*_*1*_, *R*_*2*_, … *R*_*n*_ is


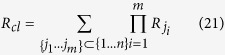


Thus, the clumped isotope signal Δ is


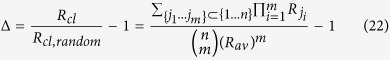


Eq. 22 is the most general equation to calculate statistical negative isotope clumping in any multi-isotope system. In the case where all ratios *R*_*i*_ are identical, *R*_*ji*_ = *R*_*av*_, the numerator becomes identical to the denominator because the number of possible subsets {j_1_, … j_m_} out of {1, … *n*} is equal to the binomial coefficient 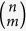 and the products reduce to the factors (*R*_*av*_)^*m*^. In this case, Δ = 0.

### More than 2 stable isotopes: ^17^O-^18^O clumping for oxygen

So far we have formally only treated molecules with two stable isotopes. Oxygen has three stable isotopes ^16^O, ^17^O and ^18^O. Clumping of heavy isotopes of the same sort (i.e., clumping of multiple ^17^O atoms or multiple ^18^O atoms in one molecule) follows the examples and general rules outlined above (see Table 1). However, it is also possible that the heavy ^17^O and ^18^O atoms clump together in one molecule.

### O_2_ and other molecules with two indistinguishable O atoms

In the case of molecular O_2_, the apparent statistical clumping signal for ^17^O-^18^O clumping was derived in Yeung *et al*.^14^. Since the isotope ratios of the individual O atoms cannot be determined individually, they are assigned the average heavy isotope ratios


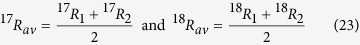


The stochastically expected (random) ratio of molecules with one ^17^O and one ^18^O isotope relative to ^16^O^16^O from this population of O atoms is


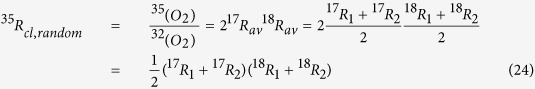


The real probability for forming ^17^O^18^O molecules from two atoms with isotope ratio ^17^*R*_*1*_, ^18^*R*_*1*_ and ^17^*R*_*2*,_^18^*R*_*2*_ is


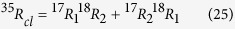


Therefore,


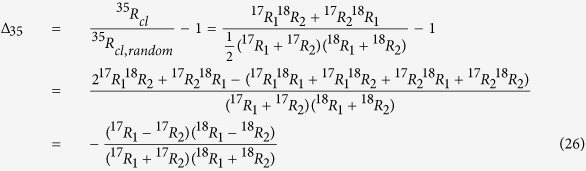


For normal mass dependent fractionation, where 
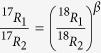
 ref. 24, with three-isotope exponent *β *≈ 0.53 ref. 25, ^17^*R*_1_ and ^18^*R*_1_ are either both smaller or both larger than ^17^*R*_2_ and ^18^*R*_2_, thus again the clumped isotope signature ^35^Δ is always <0 ( = 0 if ^i^R_1_ and ^i^R_2_ are identical). This can also be shown by inserting the mass dependent fractionation relation as follows:


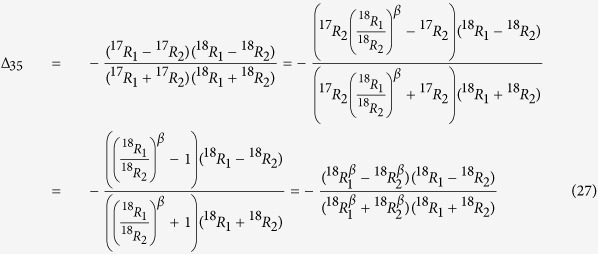


**Figure 4:**
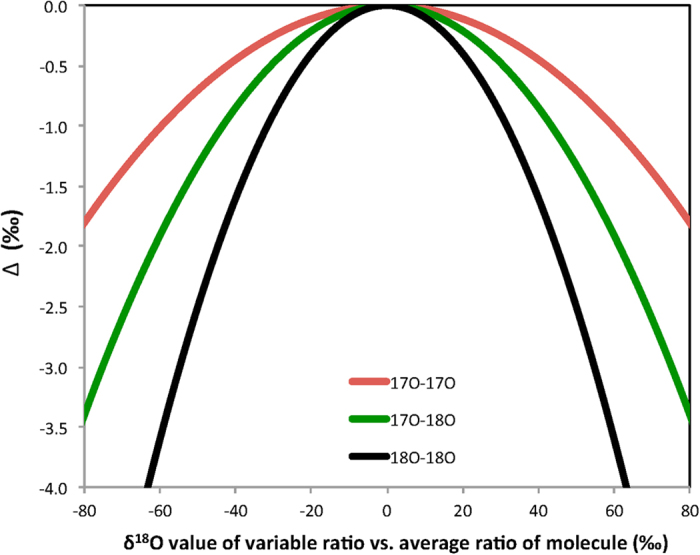
^17^O-^17^O, ^17^O-^18^O and ^18^O-^18^O clumping in O_2_ as function of the δ^18^O value of the variable isotope ratio versus the average isotope ratio in the molecule. Mass dependent fractionation according to 
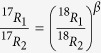
 with three-isotope exponent *β* = 0.53 ref. 25 is assumed.

As the atoms do not need to share the same bond to generate statistical isotope clumping, the equation also applies to ^17^O-^18^O clumping of other molecules with 2 O atoms (importantly ^17^O-^18^O clumping in CO_2_). The resulting negative clumping signature is shown in Fig. 4.

### Heavy isotope clumping for three indistinguishable oxygen atoms (e.g. O_3_)

When a molecule has three indistinguishable (or not distinguished) oxygen atoms, we assign the average ratios 
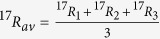
 and 
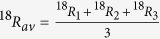
 to each of the atoms. The statistical clumping signatures for complete ^17^O or ^18^O substituted isotopocules and for ^17^O-^17^O and ^18^O-^18^O clumping can be derived according to the formalism derived above. Here we also consider the remaining combinations where ^17^O and ^18^O clump together in one molecule.

Since there are 3! = 6 different possibilities to distribute the three distinguishable atoms (^16^O, ^17^O and ^18^O) over the three positions, the stochastically expected clumping is


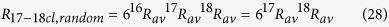


The real clumping is calculated by considering explicitly all 6 configurations of ^16^O, ^17^O and ^18^O


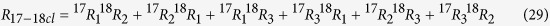


and thus


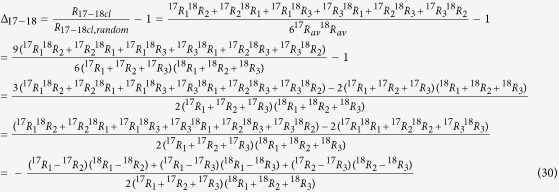


As argued above for O_2_, for normal mass dependent fractionation, ^17^*R*_i_ and ^18^*R*_i_ are either both smaller or both larger than ^17^*R*_j_ and ^18^*R*_j_, thus Δ is always < 0 (=0 if all ratios are identical). Inserting the mass dependent fractionation relation 
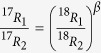
 again, Eq. 30 can be transformed to


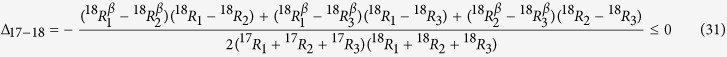


**Figure 5:**
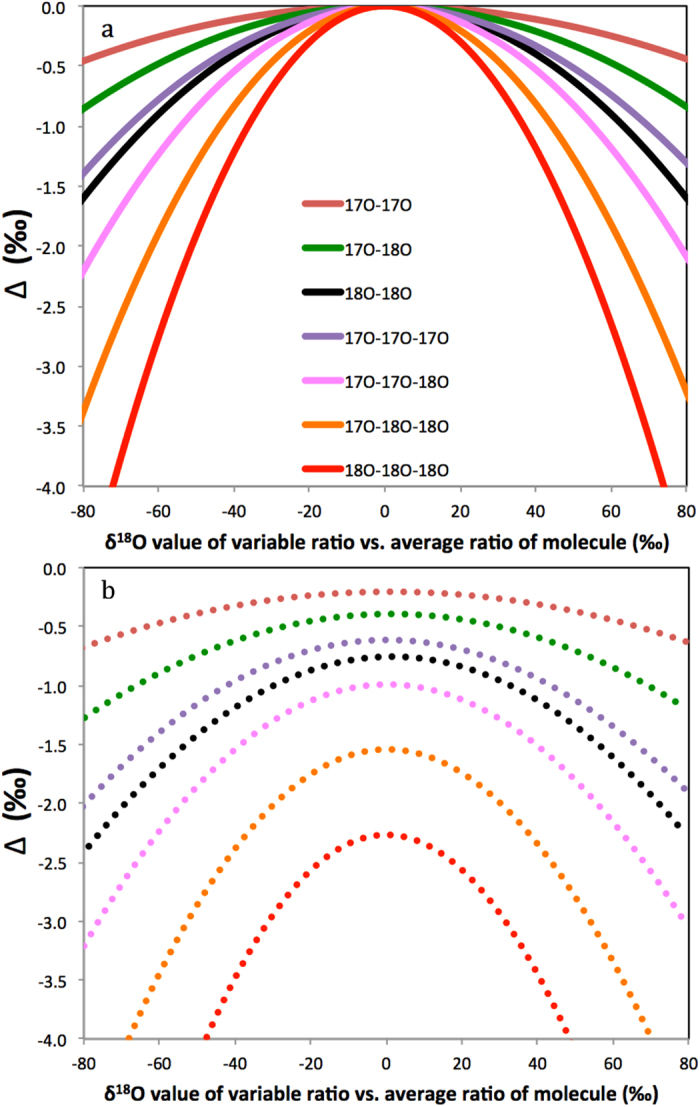
All combinations of ^17^O-^18^O heavy isotope clumping for three indistinguishable O atoms in O_3_ as function of the δ^18^O value of the variable isotope ratio versus the average isotope ratio in the molecule. The curves for clumping of a single isotope are the same as in Figures 2 and 3 (red and black curves refer to red solid curves in Figures 2 and 3, respectively). In all scenarios two isotope ratios are kept fixed and the third isotope ratio is varied. In (**a**) the two fixed isotope ratios are identical, which includes the situation that all isotope ratios are identical and Δ = 0‰, in (**b**) one of the fixed isotope ratios is 10% higher than the other. ^17^O and ^18^O ratios are linked via the mass dependent fractionation equation 
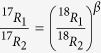
.

The corresponding values of Δ are shown in Fig. 5 together with all other possible isotope clumping combinations for ^17^O and ^18^O isotopes in O_3_. These are always calculated as the relative difference of the explicitly calculated clumped isotope ratio ^*i*^*R*_*cl*_ from the stochastically expected heavy isotope ratio ^*i*^*R*_*cl*,*random*_ (Equation 30). For example, for ^17^O-^17^O-^18^O clumping there are three possible configurations to distribute the atoms (^17^O, ^17^O and ^18^O) over the three positions, so the stochastically expected clumping is


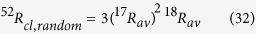


The real clumping is calculated by considering explicitly all 3 configurations


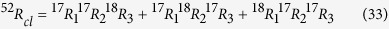


and thus


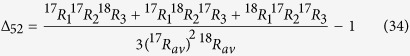


The corresponding equation for ^17^O-^18^O-^18^O clumping is easily derived by exchanging ^17^O and ^18^O.

Important examples of molecules with three oxygen atoms are O_3_ and the nitrate ion NO_3_^−^. For O_3_, the central and terminal O atoms can be distinguished with suitable techniques^2^,^26^,^27^,^28^,^29^. In these cases, statistical anti-clumping only occurs when the indistinguishable terminal O atoms are involved (Table 1). In many cases, however, the isotopic composition of O_3_ is determined without position information^30^,^31^,^32^,^33^ and in this case the atoms can be treated as effectively indistinguishable and need to be treated according to the formalism developed here.

### Statistical clumped isotope signals and the heterogeneity of the isotope ratios of indistinguishable atoms

**Figure 6:**
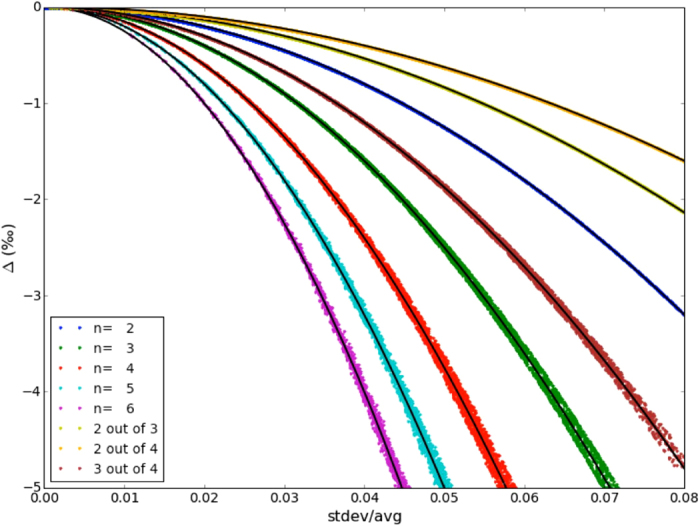
Relation between the apparent statistical heavy isotope clumping signal Δ and the relative standard deviation (stdev/average) of the involved indistinguishable atoms for randomly generated sets of isotope ratios. For each multi-isotope system, the points fall close to well-defined analytical curves (black lines) of the general form 
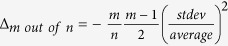
. Note that the Δ values are given in ‰, i.e. the coefficients of the quadratic term in the fit have to be divided by 1000. As an example, for 2-atomic systems (*n* = *m* = 2) the fit is Δ_*m out of n*_ = −500‰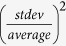.

The derivations above show that statistical combination of indistinguishable atoms in a molecule leads to apparent negative clumped isotope signals. The size of the apparent negative clumping in the molecule increases with increasing difference in isotopic composition between the individual atoms, so it may actually contain scientifically relevant information. In order to investigate this further, we created random (within a certain range) sets of isotope ratios for all multi-isotope systems with up to 5 indistinguishable atoms and calculated the apparent negative multi-isotope clumping signature Δ. Figure 6 shows that for each multi-isotope set all these random sets of isotope ratios yield Δ values that fall on distinct curves when Δ is plotted versus the relative standard deviation of the individual isotope ratios. For each of the multi-isotope clumping combinations with *m* out of *n* heavy isotopes, the curves can be parameterized as:


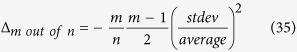


Note that the Δ values in Fig. 6 are plotted as ‰, so the factor 1/2 in Eq. 35 corresponds to 500‰ in the fit curves.

**Figure 7:**
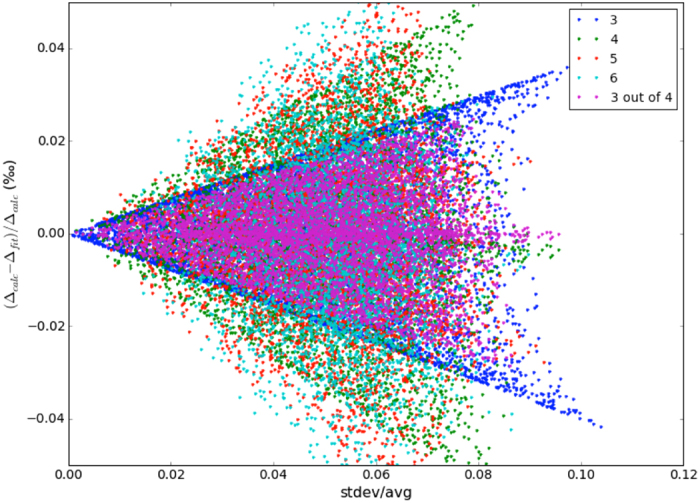
Difference between the analytical curves shown as black lines in Fig. 6 and the calculated value of Δ for the randomly generated sets of isotope ratios (colored points in Fig. 6). For each multi-isotope system, the outer envelope of the points gives the error within which the apparent statistical isotope clumping can be predicted for a certain set of isotope ratios when the relative standard deviation (stdev/average) of the isotope ratios of the indistinguishable atoms is known.

In the case of m = 2 (2 heavy isotopes in any multi-isotope system) the fit is perfect, but for more heavy atoms clumping in one molecule there is some scatter around these fit lines. This originates from the fact that there is no fixed analytical relation between the arithmetic and geometric means. Figure 7 shows the relative deviation of the explicitly calculated Δ values and the approximation using Eq. 35 for the randomly chosen sets of isotope ratios with known standard deviation and average values. The outer envelopes of the point clouds for each multi-isotope system define the error with which the statistical clumped isotope signal Δ in a molecule can be predicted from Eq. 35 when the standard deviation and the mean of the individual isotope ratios are known.

Scientifically, the opposite relation


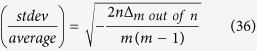


is more attractive, since the isotopic variability among indistinguishable atoms in a molecule is usually not known, but Δ may be measurable^14^. This means that measurement of the statistical clumped isotope anomaly Δ could provide a novel tracer to determine the heterogeneity (quantified by the standard deviation) of the isotopic pools of indistinguishable atoms in a molecule. For example, in the 2-isotope system O_2_, a Δ value of 1.5‰ below the thermodynamically expected value as measured by Yeung *et al*.^14^ would indicate a relative standard deviation in the isotope ratios of the two O atoms of about 5.5% (Fig. 6, blue curve).

**Figure 8:**
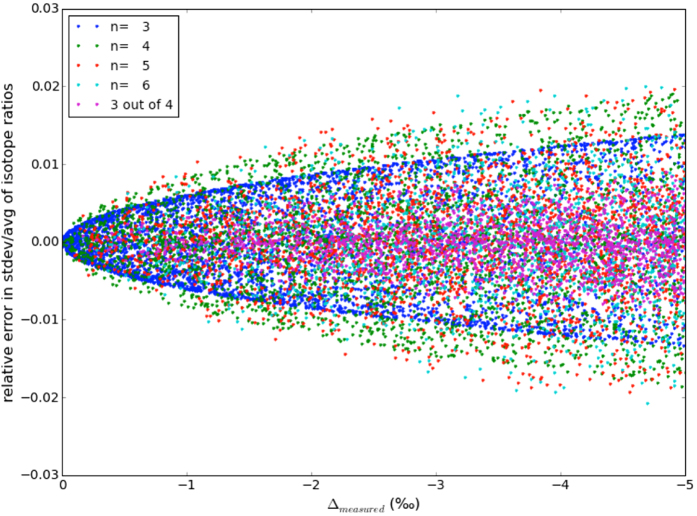
Relative difference of the heterogeneity (relative standard deviation) of the isotope ratios of indistinguishable atoms calculated using Eq. 38 from the true heterogeneity of the randomly chosen sets of isotope ratios. For each multi-isotope system, the outer envelope of the points gives the error within which the heterogeneity of indistinguishable atoms in a certain multi-isotope system can be calculated from the apparent statistical isotope clumping signal Δ.

Figure 8 shows the relative error that is made when Eq. 36 is used to calculate the relative standard deviation of the isotope ratios from the Δ value for randomly chosen sets of (known) isotope ratios. The outer envelope for each isotope system quantifies the error with which 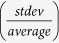 for a group of indistinguishable atoms can be derived from Δ. Above Δ values of −1.5‰ the relative error in 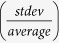 is generally below 1%, and above Δ values of −5‰ the relative error is still only 2%. Thus, whereas we are not able to measure the isotopic composition of individual indistinguishable atoms, the apparent statistical isotope clumping provides a means to obtain information about the heterogeneity of the isotope ratios with quite good precision.

## Conclusions

The statistical combination of indistinguishable atoms with different isotope ratios in a molecule always leads to apparent negative clumped isotope signals. We emphasize the term apparent, because this signal does not relate to a physical negative clumping process. The underlying reason is that in the calculation of the stochastic reference value for calculating Δ, the actual isotopic composition at each individual atom position is replaced by the average of the isotopic composition of all indistinguishable atoms (which is similar bulk isotopic composition of the molecule for small isotope ratios). Thus the apparent statistical Δ is by nature an artifact originating from our limitation to measure the isotope ratios of indistinguishable atoms. Using the formalism presented in this paper, this apparent statistical clumping signal can be calculated for any multi-isotope system.

We have calculated here the pure apparent statistical heavy isotope clumping values. In nature these apparent clumping signals will always occur in combination with thermodynamic heavy isotope clumping and possible other kinetic isotope effects that lead to clumped isotope anomalies (Yeung^21^. The statistical clumped isotope signatures will *always* occur whenever two or more indistinguishable atoms clump together in a molecule. For isotope heterogeneities of a few percent, the effect is of the same order of magnitude as the thermodynamic effects in many molecules. Thus, it is important to take these effects into consideration when interpreting isotopic clumping of indistinguishable atoms in nature. Furthermore, when the statistical clumping signature can be separated from other contributions, its magnitude provides quantitative information on the heterogeneity of the isotopic composition of the indistinguishable atoms in a molecule.

## Additional Information

**How to cite this article**: Röckmann, T. *et al*. Statistical clumped isotope signatures. *Sci. Rep.* **6**, 31947; doi: 10.1038/srep31947 (2016).

## Supplementary Material

Supplementary Information

## Figures and Tables

**Table 1:** Combinations of dual heavy isotope clumping in selected atmospheric compounds and information whether the corresponding clumped isotope signatures are affected by apparent statistical clumped isotope effects or not.

Compound	Isotope Combinations		
H_2_	^2^H^2^H		Anti-clumping
O_2_	^18^O^18^O	^17^O^17^O	Anti-clumping
	^17^O^18^O		Anti-clumping
N_2_	^15^N^15^N		Anti-clumping
H_2_O	^1^H^2^H^18^O	^1^H^2^H^17^O	No effect
	^2^H^2^H^16^O		Anti-clumping
CO	^13^C^18^O	^13^C^17^O	No effect
CO_2_	^13^C^18^O^16^O	^13^C^17^O^16^O	No effect
	^12^C^18^O^18^O	^12^C^17^O^17^O	Anti-clumping
	^12^C^17^O^18^O		Anti-clumping
N_2_O	^14^N^15^N^18^O	^14^N^15^N^17^O	No effect
	^15^N^15^N^16^O		Anti-clumping*
CH_4_	^13^CH_3_^2^H		No effect
	^12^CH_2_^2^H _2_		Anti-clumping
O_3_	^18^O^16^O^18^O	^17^O^16^O^17^O	Anti-clumping
	^16^O^18^O^18^O	^16^O^17^O^17^O	Anti-clumping**
	^17^O^16^O^18^O		Anti-clumping
	^16^O^17^O^18^O	^16^O^18^O^17^O	Anti-clumping**
NO_2_	^18^O^15^N^16^O	^17^O^15^N^16^O	No effect
	^18^O^14^N^18^O	^17^O^14^N^17^O	Anti-clumping
	^17^O^14^N^18^O		Anti-clumping
C_2_H_6_	^13^C^12^C^2^H^1^H_5_		No effect
	^13^C^13^C^1^H_6_		Anti-clumping
	^12^C^12^C ^2^H_2_^1^H_4_		Anti-clumping

^*^If the N atom positions cannot be individually determined.

^**^If symmetric and asymmetric O atoms cannot be individually determined.

## References

[b1] Kaiser J., Röckmann T. & Brenninkmeijer C. A. M. Assessment of ^15^N^15^N^16^O as a tracer of stratospheric processes. Geophys. Res. Lett. 30, 10.1029/2002GL016073 (2003).

[b2] Janssen C., Guenther J., Krankowsky D. & Mauersberger K. Relative formation rates of ^50^O_3_ and ^52^O_3_ in ^16^O-^18^O mixtures. J. Chem. Phys. 111, 7179–7182 (1999).

[b3] Mauersberger K., Erbacher B., Krankowsky D., Günther J. & Nickel R. Ozone isotope enrichment: Isotopomer-specific rate coefficients. Science 283, 370–372 (1999). 9888849 10.1126/science.283.5400.370

[b4] Hauck R. D. & Bouldin D. R. Distribution of Isotopic Nitrogen in Nitrogen Gas During Denitrification. Nature 191, 871–872 (1961).

[b5] Mroz E. J. . Detection of Multiply Deuterated Methane in the Atmosphere. Geophys. Res. Lett. 16, 677–678, 10.1029/Gl016i007p00677 (1989).

[b6] Eiler J. M. & Schauble E. ^18^O^13^C^16^O in Earth’s atmosphere. Geochim. Cosmochim. Acta 68, 4767–4777, 10.1016/j.gca.2004.05.035 (2004).

[b7] Yeung L. Y., Young E. D. & Schauble E. A. Measurements of ^18^O-^18^O and ^17^O-^18^O in the atmosphere and the role of isotope-exchange reactions. J. Geophys. Res. 117, D18306, 18310.11029/12012JD017992 (2012).

[b8] Eiler J. M. . A high-resolution gas-source isotope ratio mass spectrometer. Int J. Mass Spect. 335, 45–56 (2013).

[b9] Ono S. . Measurement of a Doubly Substituted Methane Isotopologue, ^13^CH_3_D, by Tunable Infrared Laser Direct Absorption Spectroscopy. Anal. Chem. 86, 6487–6494, 10.1021/ac5010579 (2014). 24895840

[b10] Stolper D. A. . Formation temperatures of thermogenic and biogenic methane. Science 344, 1500–1503, 10.1126/science.1254509 (2014). 24970083

[b11] Stolper D. A. . Combined ^13^C-D and D-D clumping in methane: Methods and preliminary results. Geochim. Cosmochim. Acta 126, 169–191, 10.1016/j.gca.2013.10.045 (2014).

[b12] Stolper D. A. . Distinguishing and understanding thermogenic and biogenic sources of methane using multiply substituted isotopologues. Geochim. Cosmochim. Acta 161, 219–247, 10.1016/j.gca.2015.04.015 (2015).

[b13] Wang D. T. . Nonequilibrium clumped isotope signals in microbial methane. Science 348, 428–431, 10.1126/science.aaa4326 (2015). 25745067

[b14] Yeung L. Y., Ash J. L. & Young E. D. Biological signatures in clumped isotopes of O_2_. Science 348, 431–434, 10.1126/science.aaa6284 (2015). 25908819

[b15] Magyar P. M., Orphan V. J. & Eiler J. M. Insights into Mechanisms of Nitrous Oxide Generation from Measurement of Nine N_2_O Isotopologues Goldschmidt Abstracts, 2015, 1970 (2015).

[b16] Urey H. C. The thermodynamic properties of isotopic substances J. Chem. Soc. 562–581 (1947). 20249764 10.1039/jr9470000562

[b17] Bigeleisen J. & Mayer M. G. Calculation of equilibrium constants for isotopic exchange reactions. J. Chem. Phys. 15, 261–267 (1947).

[b18] Richet P., Bottinga Y. & Javoy M. A review of hydrogen, carbon, nitrogen, oxygen, sulphur, and chlorine stable isotope fractionation among gaseous molecules. Ann. Rev. Earth Planet. Sci. 5, 65–110 (1977).

[b19] Wang Z. G., Schauble E. A. & Eiler J. M. Equilibrium thermodynamics of multiply substituted isotopologues of molecular gases. Geochim. Cosmochim. Acta 68, 4779–4797 (2004).

[b20] Eiler J. M. “Clumped-isotope” geochemistry—The study of naturally-occurring, multiply-substituted isotopologues. Earth Planet. Sci. Lett. 262s, 309–327 (2007).

[b21] Yeung L. Y. Combinatorial effects on clumped isotopes and their significance in biogeochemistry. Geochim. Cosmochim. Act, 10.1016/j.gca.2015.1009.1020 (2016).

[b22] Brenninkmeijer C. A. M. & Röckmann T. Mass spectrometry of the intramolecular nitrogen isotope distribution of environmental nitrous oxide using fragment-ion analysis. Rap. Commun. Mass Spectrom 13, 2028–2033 (1999). 10.1002/(SICI)1097-0231(19991030)13:20<2028::AID-RCM751>3.0.CO;2-J 10510416

[b23] Toyoda S. & Yoshida N. Determination of nitrogen isotopomers of nitrous oxide on a modified isotope ratio mass spectrometer. Anal. Chem. 71, 4711–4718 (1999).

[b24] Kaiser J., Röckmann T. & Brenninkmeijer C. A. M. Contribution of mass-dependent fractionation to the oxygen isotope anomaly of atmospheric nitrous oxide. J. Geophys. Res. 109, D03305, 10.1029/2003JD004088 (2004).

[b25] Young E. D., Galy A. & Nagahara H. Kinetic and equilibrium mass-dependent isotope fractionation laws in nature and their geochemical and cosmochemical significance. Geochim. Cosmochim. Acta 66, 1095–1104 (2002).

[b26] Vicars W. C., Bhattacharya S. K., Erbland J. & Savarino J. Measurement of the ^17^O-excess (Δ^17^O) of tropospheric ozone using a nitrite-coated filter. Rapid Commun. Mass Spectrom. 26, 1219–1231, 10.1002/Rcm.6218 (2012). 22499198

[b27] Janssen C. Intramolecular isotope distribution in heavy ozone (^16^O-^18^O-^16^O and ^16^O-^16^O-^18^O). J. Geophys. Res. 110, D08308, 08310.01029/02004JD005479 (2005).

[b28] Larsen R. W., Larsen N. W., Nicolaisen F. M., Sorensen G. O. & Beukes J. A. Measurements of ^18^O-enriched ozone isotopomer abundances using high-resolution Fourier transform far-IR spectroscopy. J. Mol. Spectrosc. 200, 235–247 (2000). 10708536 10.1006/jmsp.2000.8059

[b29] Johnson D. G., Jucks K. W., Traub W. A. & Chance K. V. Isotopic composition of stratospheric ozone. J. Geophys. Res. 105, 9025–9031 (2000).

[b30] Shaheen R., Janssen C. & Röckmann T. Investigations of the photochemical isotope equilibrium between O_2_, CO_2_ and O_3_. Atmos. Chem. Phys. 7, 495–509 (2007).

[b31] Johnston J. C., Röckmann T. & Brenninkmeijer C. A. M. CO_2_+O(^1^D) isotopic exchange: Laboratory and modeling studies. J. Geophys. Res. 105, 15213–15229 (2000).

[b32] Früchtl M., Janssen C. & Röckmann T. Experimental study on isotope fractionation effects in visible photolysis of O_3_ and in the O+O_3_ odd oxygen sink reaction. J. Geophys. Res. 120, 4398–4416, 10.1002/2014JD022944 (2015).

[b33] Chakraborty S. & Bhattacharya S. K. Oxygen isotopic fractionation during UV and visible light photodissociation of ozone. J. Chem. Phys. 118, 2164–2172 (2003).

