# Supplementary material for: Statistical clumped isotope signatures
Source: Sci Rep. 2016 Aug 18;6:31947. doi: 10.1038/srep31947 (PMC4989146; doi:10.1038/srep31947)
Supplement: Supplementary Information [file srep31947-s1.pdf]

Statistical clumped isotope signatures

T. Röckmann[1,*], M.E. Popa[1], M.C. Krol[1,2,3] and M.E.G. Hofmann[1]

[1]Institute for Marine and Atmospheric research Utrecht (IMAU), Utrecht University, Utrecht, The Netherlands
[2]Wageningen University, Wageningen, Netherlands.
[3]SRON Netherlands Institute for Space Research, Utrecht, Netherlands.
[*] corresponding author; email: t.roeckmann@uu.nl

Supplementary Information

Difference between using average and bulk isotope ratios

The origin of apparent clumped isotope signatures is that one constant value is assigned to the isotope ratios of all indistinguishable atoms in a molecule for calculation of the stochastically expected clumped isotope ratio. This value ends up as reference ratio in the denominator of the definition of the clumped isotope signature Δ (e.g. Eq. 12 or 16). The conventional choice is to use the bulk isotope ratio R_{bulk} as the reference, but we have used the average isotope ratio R_{av} in the equations.

The relation between R_{bulk} and R_{av} can be illustrated by writing up explicitly the definition of R_{bulk}, where we add up the number of light and heavy atoms, respectively, in all isotopologues of the molecule. For the case of 2 ^{15}N atoms in N_2, this is

$$
\begin{aligned}
{}^{15}R_{bulk} &= \frac{\text{number of } {}^{15}\text{N atoms in the sample}}{\text{number of } {}^{14}\text{N atoms in the sample}} \\
&= \frac{2\left[{}^{15}\text{N}_1{}^{15}\text{N}_2\right]+\left[{}^{15}\text{N}_1{}^{14}\text{N}_2\right]+\left[{}^{14}\text{N}_1{}^{15}\text{N}_2\right]}{2\left[{}^{14}\text{N}_1{}^{14}\text{N}_2\right]+\left[{}^{15}\text{N}_1{}^{14}\text{N}_2\right]+\left[{}^{14}\text{N}_1{}^{15}\text{N}_2\right]} \\
&= \frac{2\left[{}^{15}\text{N}_1\right]\left[{}^{15}\text{N}_2\right]+\left[{}^{15}\text{N}_1\right]\left[{}^{14}\text{N}_2\right]+\left[{}^{14}\text{N}_1\right]\left[{}^{15}\text{N}_2\right]}{2\left[{}^{14}\text{N}_1\right]\left[{}^{14}\text{N}_2\right]+\left[{}^{15}\text{N}_1\right]\left[{}^{14}\text{N}_2\right]+\left[{}^{14}\text{N}_1\right]\left[{}^{15}\text{N}_2\right]} \\
&= \frac{2\,{}^{15}R_1\,{}^{15}R_2+{}^{15}R_1+{}^{15}R_2}{2+{}^{15}R_1+{}^{15}R_2} \approx \frac{{}^{15}R_1+{}^{15}R_2}{2} = {}^{15}R_{av}
\end{aligned}
\qquad (1)
$$

The quantities in brackets are abundances of the respective isotopologues and isotopes. Since we want to calculate the stochastic reference, we have used the assumption of random isotope distribution, i.e., that the abundance of certain isotopologues is the product of the abundance of the contributing isotopes. It can now be seen easily that the transition from R_{bulk} to R_{av} is made by neglecting the higher order terms in R_i both in the numerator (higher than order 1) and denominator (higher than order 0), and that these terms are actually the same in numerator and denominator. Similar equations can be derived for multiple-isotope systems, where all higher order terms in R_i have to be neglected in the approximation. Thus, the approximation will be very good for R_i << 1 and the difference between R_{bulk} and R_{av} will increase with increasing R_i.

One advantage of using R_{av} as reference is that it allows for a straightforward "geometric" interpretation of the statistical clumped isotope anomalies in terms of geometric versus arithmetic means. Another advantage is that the apparent Δ value does not depend on the absolute isotope ratio when R_{av} is chosen as reference, whereas it does depend on the isotope ratio when R_{bulk} is used. This is shown for two examples of isotopic heterogeneity and various multi-isotope systems in Figure S1. The figure shows calculations of the apparent clumping signatures Δ (as used in this paper) and Δ_{bulk} (when R_{bulk} is used as reference) as a function of R_{av}. At each value of R_{av}, the isotope ratio of one of the atoms is 5% heavier (blue, black lines) and 10% heavier (red, green lines) than the other isotope ratios, but this difference is held constant as R_{av} varies. For Δ, the lines are constant because in fact they represent

one individual point on a certain curve in Figure 2 or 3 for different values of R_{av}. However, Δ_{bulk} shows a strong decrease as R_{av} approaches 1, and it even changes sign for R_{av} >1.

Figure S2 shows the absolute and relative differences between the two definitions. The absolute difference between Δ and Δ_{bulk} depends on the average isotope ratio and on the heterogeneity of the isotope ratios. For a difference of 10‰ in the isotope ratio of one atom (factor 1.01) the differences are <0.01‰ for all multi-isotope systems up to values of about R_{av} = 0.1. Larger differences would occur for elements with larger heavy isotope ratios such as Cl or Br. For a difference of 30‰ in one heavy isotope ratio (factor 1.03), the difference between Δ and Δ_{bulk} increases but is still below 0.01‰ at typical isotope ratios of the light elements (HCNO). If one isotope ratio is 100‰ higher than the others (factor 1.1), the difference is already of the order of 0.05‰ for the isotope ratio of ^{13}C (1.1%). However, also the absolute values Δ and Δ_{bulk} depend on the heterogeneity (multiplication factor) and Δ_{bulk} furthermore depends on R_{av} (Figure S2a). Therefore, the different lines depicting the absolute difference collapse on one line for the relative difference between Δ and Δ_{bulk} for all multi-isotope systems (Figure S2b). The relative difference between Δ and Δ_{bulk} is approximately 2 times R_{av} in the range of natural heavy isotope values.

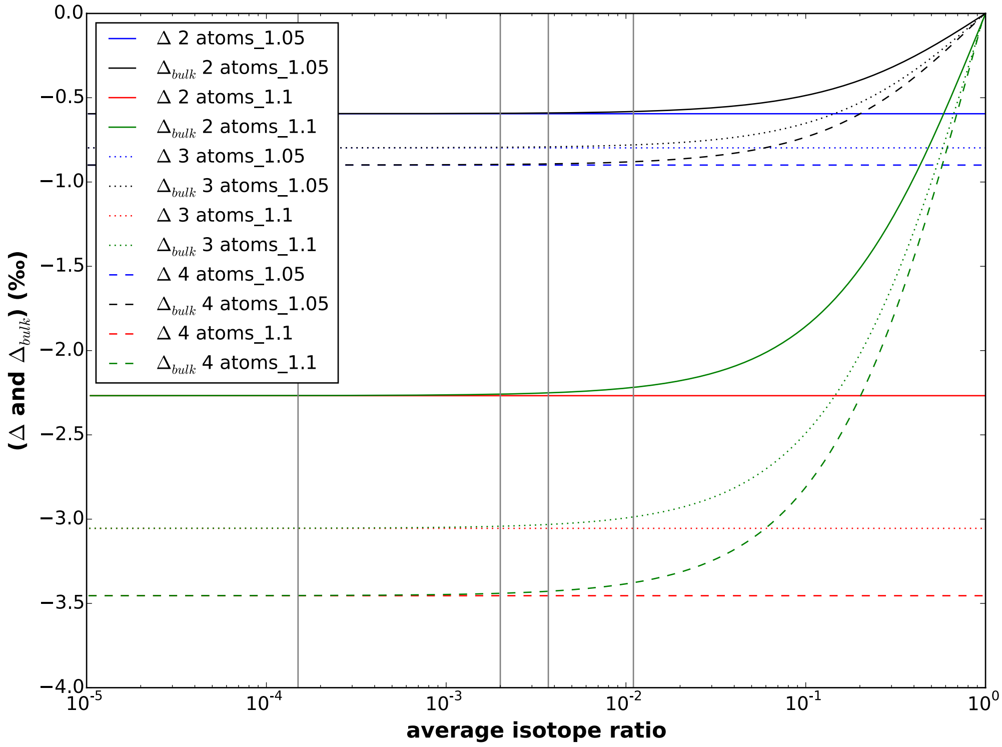

Figure S1: Apparent clumped isotope values Δ (blue and red lines) and Δ_{bulk} black and green lines) as a function of the average isotope ratio of all atoms for two cases where the isotope ratio of one of the atoms is 5% heavier (blue, black lines) and 10% heavier (red, green lines) than the other isotope ratios. Solid lines depict two-isotope systems, dotted lines three-isotope systems and dashed lines 4-isotope systems. These lines represent one individual point on a certain curve in Figure 2 or 3 for different values of R_{av}. Δ as defined in this paper is independent of R_{av}, whereas Δ_{bulk} shows a strong decrease as R_{av} approaches 1 (Δ_{bulk} even changes sign for R_{av} > 1, not shown). The vertical grey lines indicate the typical terrestrial abundances for the heavy-to-light isotope ratios of the light elements of $^{13}C/^{12}C$ $=1.1*10^{-2}$, $^{15}N/^{14}N$ $=3.7*10^{-3}$, $^{18}O/^{16}O$ $=2*10^{-3}$ and $^{2}H/^{1}H$ $=1.5*10^{-4}$.

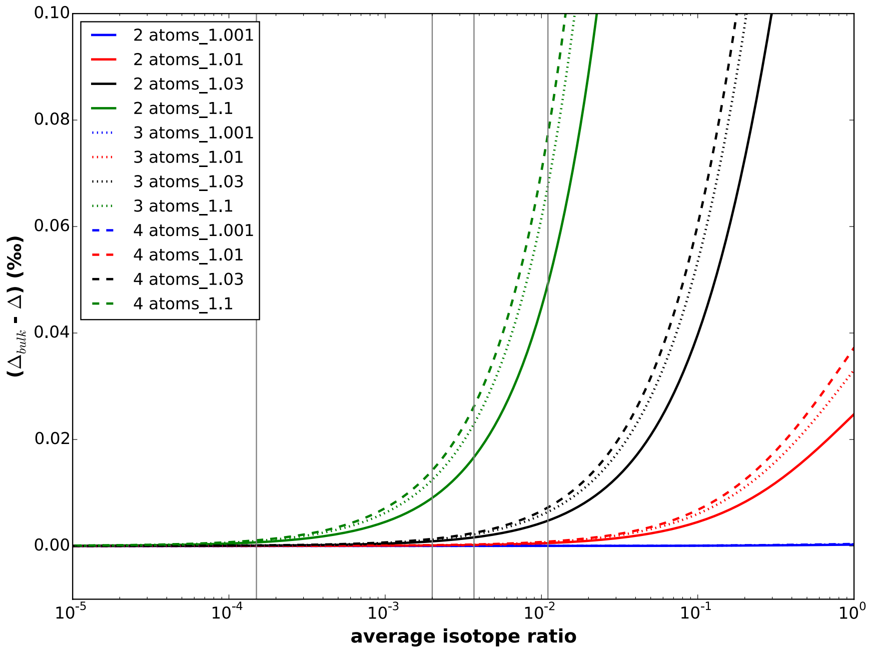

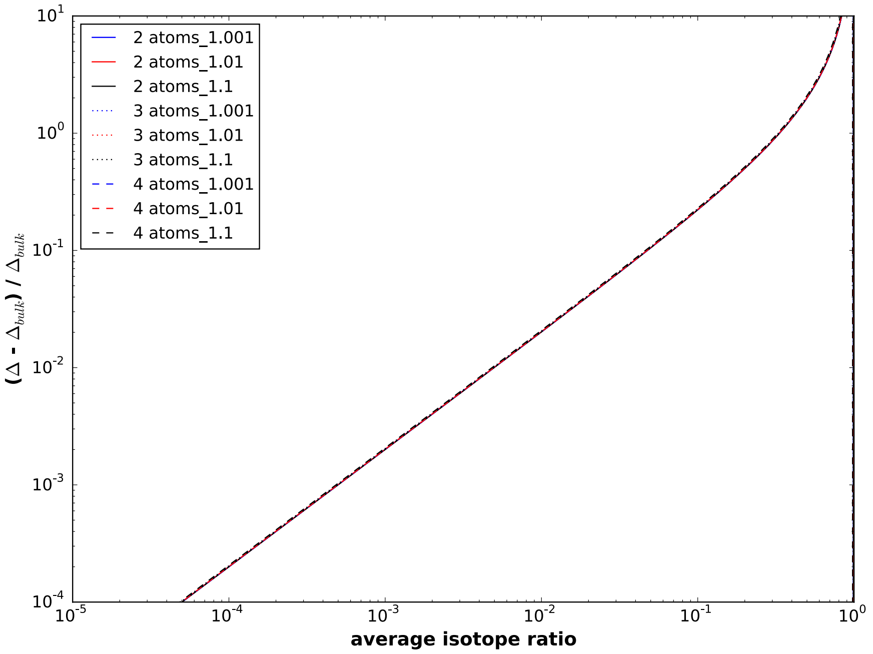

Figure S2: Absolute (top) and relative (bottom) difference between Δ_{bulk} and Δ (in ‰) as a function of the average isotope ratio of all atoms. Δ_{bulk} is the apparent clumped isotope signal when assigning the bulk isotope composition to the individual isotope ratios and Δ is the same signal when assigning the average isotopic composition. For these calculations, the isotope ratio was varied over five

orders of magnitude and at each value one of the isotope ratios was multiplied by a factor (1.001, 1.01, 1.1) as indicated in the legend to create a certain isotope heterogeneity. Solid lines depict two-isotope systems, dotted lines three-isotope systems and dashed lines 4-isotope systems. The absolute difference between the two definitions depends on the average isotope ratio and on the heterogeneity, but the relative differences are very similar for all systems. They do actually start deviating more when the isotopic heterogeneity increases even further (not shown), but cases with isotope differences of much more than 100‰ will be rare in nature. The vertical grey lines indicate the typical terrestrial abundances for the heavy-to-light isotope ratios of the light elements of $^{13}C/^{12}C = 1.1*10^{-2}$, $^{15}N/^{14}N = 3.7*10^{-3}$, $^{18}O/^{16}O = 2*10^{-3}$ and $^{2}H/^{1}H = 1.5*10^{-4}$.
